# What is the best option to manage the bedside teaching for Neurology Clerkship -- Demonstration, Simulation or WeChat teaching?

**DOI:** 10.15694/mep.2020.000286.1

**Published:** 2020-12-22

**Authors:** Hongyan Zhou, Li Feng, Yaru Lu, Jingjing Li, Xunsha Sun, Cunzhou Shen, Weixi Zhang, Ling Chen, Dan Xu, Ming Kuang, Huiyu Feng

**Affiliations:** 1 The First Affiliated Hospital of Sun Yat-sen University; 2 Curtin University Curtin Medical School

**Keywords:** Neurological clerkship, Bedside teaching, Demonstration, Simulation, WeChat-assisted teaching

## Abstract

This article was migrated. The article was marked as recommended.

**Background:** Neurological clerkship is an important but challenging part of the neurology curriculum, and bedside teaching is essential for clerks to integrate complex theories and skills into practice.

**Objective:** This study aimed to investigate the three bedside teaching methods as modified bedside demonstration, simulation via standard patients as well as WeChat-assisted teaching during neurological clerkship, in order to identify the optimal method.

**Design:** The4 ^th^-year medical students were enrolled during their neurological clerkship. Bedside teaching on topics of acute ischemic stroke, acute myelitis and myasthenia gravis were delivered in a random order of demonstration, simulation and WeChat-assisted teaching. A questionnaire was assigned to each participant at the end of the two-week clerkship to rank the three methods based on their general impression, as well as detailed assessment of clinical abilities and attitudes.

**Results:** A total of 112 clerks were enrolled and 98 were eligible for analysis. For both general and overall ranking, simulation was the most approved bedside teaching method while WeChat-assisted teaching took the least approval among the three. A majority of participants preferred simulation because of the significant improvement on interpretation of diseases, interest in neurology, diagnostic skills, clinical skills, communication skills, empathy and protection of privacy (P＜0.05). Demonstration was considered to benefit performance in examination (P=0.009). The ranking of the three methods revealed different consistency between general impression and detailed assessment. More participants tended to underestimate themselves in simulation but to overestimate themselves in WeChat-assisted teaching (P=0.000).

**Conclusions:** Simulation outweigh WeChat-assisted teaching and demonstration by promoting clinical skills, as well as inspiring students’ academic interest, compassion and empathy in both the general ranking and the overall ranking. Clerks were tended to underestimate their clinical competence in simulation but overestimate themselves in WeChat-assisted teaching.

## Introduction

Patients with neurological diseases are usually first assessed by general practitioners or internal medicine physicians before referred to neurologists. Therefore, it is essential for every graduating medical student to be able to manage the assessment and treatment of common neurological diseases ( Roze *et al.*, 2018). However, neurology is considered as one of the most difficult subjects among medicine due to its complex theories, semiology, physical examination and diagnostic skills, which are hard to master ( Jozefowicz, 1994; Pakpoor *et al.*, 2014). In the 5-year undergraduate clinical medicine program in China, clerkship of eight to twelve months since the middle 4 ^th^ year is scheduled for students who have completed the pre-clinical curriculum and start the clinical curriculum to learn navigating the responsibilities of clinicians ( Perrella *et al.*, 2018). Clerkship is a very important beginning of the clinical curriculum to integrate the theoretical textbook knowledge into clinical practical skills, setting off the blueprint of medical career. Bedside teaching takes an essential role in the neurological clerkship. It provides support for students to overcome neurophobia by supervised clinical practice ( Albert *et al.*, 2015). However, extra stress and fear may be generated during bedside practice, especially when students deal with the patients with impaired consciousness or movement disorders, subsequently leading to mounted intrinsic learning load and poor learning outcomes ( Young *et al.*, 2014; Mancinetti, Guttormsen and Berendonk, 2019).

Previously, we have improved the traditional bedside teaching of neurological clerkship by implementing the in-classroom discussion and illustration after bedside demonstration rather than only brief explanation at bedside ( Jingjing *et al.*, 2017). The modified bedside demonstration method has been proven to be more effective in terms of the interpretation of theories and the grasp of clinical skills. However, it has been noticed with disadvantages including less active engagement of students, shortage of typical cases and limited patients’ collaboration. More importantly, the stress associated with face-to-face practice still exists and elicits negative effect on learning. A more advanced method of bedside teaching should be explored to decrease the students’ anxiety when they initiate the first step of being real doctors.

One of the most popular alternatives to bedside demonstration is simulation. Recent studies have shown that there has been increasing interest of using simulated patients in medical education. Learning via simulated patients facilitates the achievement of specific learning objectives without the clinical-decision-making pressure in front of real patients. The students could achieve the goal of clinical training via standardized and repetitive practice without worrying about annoying or hurting patients ( Galtrey *et al.*, 2018; Wijdicks and Hocker, 2018).

Implementation of social mediums is another way to alleviate the pressure resulting from face-to-face communication. For example, WeChat, the most popular mobile social medium in China nowadays, allows free and convenient communication ( Sun *et al.*, 2016; Zeng *et al.*, 2016b). Students can discuss cases within WeChat groups as well as communicate with teachers privately, avoiding embarrassment of questioning in front of classmates or patients. Additionally, data could be transmitted via WeChat in almost all common formats, including words, excels, PDF files, images, videos and internet links. It has been proven to achieve better student-centered teaching in the biochemistry and cellular biology courses as an active-learning teaching tool ( Wang *et al.*, 2017).

We are planning to investigate the effect of modified bedside demonstration, simulated patient teaching and WeChat-assisted bedside teaching in terms of the general impression and detailed assessment of subjects in order to determine the optimal method for bedside training in neurological clerkship.

## Methods

### 2.1 Participants

Graduating students in their 4 ^th^ year of medical school were enrolled into this study from Sep 2017 to May 2018 during their neurological clerkship. The teacher in charge of each course was strictly related to his or her specified topic and the simulated patient was role-played by a trained neurologist.

### 2.2 Curriculum design

**Figure 1.**
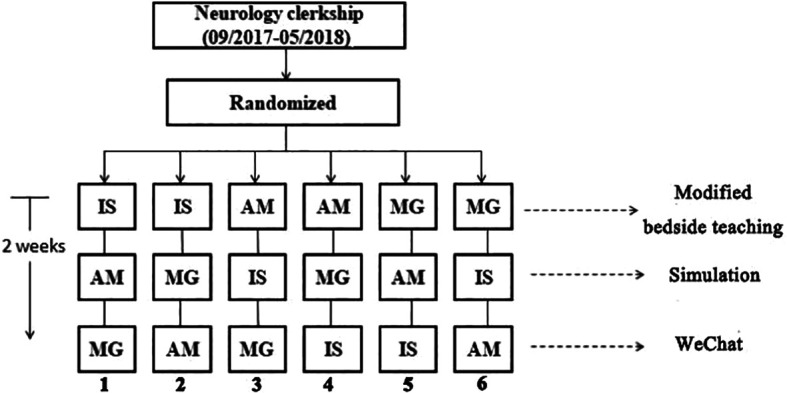
Flow chart of the study. IS: ischemic stroke; AM: acute myelitis; MG: myasthenia gravis.

The teaching units were established with various orders of topics including acute ischemic stroke, acute myelitis and myasthenia. Every group of students was assigned with a randomized number from 1 to 6, representing the different teaching units. For each group, the teaching unit was completed within 2 weeks in the order of modified bedside demonstration, simulated patient teaching and WeChat-assisted teaching ( Figure 1). The learning outcome was that every participant should be capable of managing diagnosis of the three diseases and tackling them for the first step.

*Modified bedside demonstration:* The modified bedside demonstration began with bedside demonstration of taking history and physical examination, followed by an in-classroom group discussion on diagnosis and treatment, as well as a brief summary by the teacher.

*Simulated patient teaching:* The simulated patient was role-played by a neurologist who had been trained (Supplementary File 1). Each student was required to complete at least one of the following tasks with the simulated patient independently: history taking, neurological examination (usually divided into 2-3 sections), explaining the diagnosis and treatment to the patient (1-2 sections). The tasks were randomly assigned to students. Teachers observed and assessed the students’ performance, and gave instant feedback during the simulation.

*WeChat-assisted teaching:* A WeChat groupwas established for every batch of students at the beginning of their neurological internship, and all students were advised to add their teachers into their personal WeChat lists to facilitate instant communication. Materials about the selected cases were assigned in the WeChat group the day before class. Students were asked to propose at least two questions about the case via WeChat. Bedside teaching was then carried out with the teacher’s bedside demonstration and the students’ trial under supervision. Finally, the teacher summarized the students’ performance and answered some of the questions pre-collected by WeChat in classroom. Other questions were replied privately via WeChat after class. Supplemental learning materials including updated guidelines and teaching videos were also shared via WeChat after class in order to direct self-learning. Besides, the teachers were always available on WeChat during the clerkship in case the clerks had other questions.

### 2.3 Measurement of outcomes

A questionnaire containing ten items was distributed to each subject at the end of the 2-week neurological clerkship. This survey was designed to evaluate the teaching effectiveness by the views of students. Students were asked to rank the three teaching methods according to their general impression and then the detailed items about teaching effectiveness. The questionnaire was completed by subjects voluntarily and anonymously. All the questionnaire was recorded except the uncompleted ones.

The general ranks of the three methods were summarized to determine the students’ preference in terms of the general impression of the methods. Then, the total number of different ranks of respective methods was counted for each item to reveal the students’ attitude to specific aspects. The comparison of the number of respective ranks was carried out to reveal individual’s preference for respective methods in specific aspects.

Further, the overall distribution of ranks was calculated for every method according to the total number of each rank so that the new comprehensive ranks were generated and defined as the overall ranks. The overall ranks were compared with the general ranks to reveal the change of students’ preference for the three methods.

Moreover, new ranks of the three methods derived from the detailed ranks was calculated as the objective ranks for every questionnaire and matched with the general ranks of the same questionnaire. The objective ranks represented the ranks of the three methods after careful thinking of students with the detailed tips of academic assessment, while the general ranks of methods were defined as the subjective ranks. The difference between subjective ranks and objective ranks was considered to represent the gap between subjective impression and objective assessment.

### 2.4 Statistics

Random number from 1 to 6 was generated by SPSS 17.0. The total number of each rank for respective method in general ranking was counted directly. The total number was counted separately according to different ranks of each method for individual items and the distribution of ranks was compared for each item. In addition, for each method, the overall rank was determined by comparing the sum of each rank. Besides, the objective ranks of the three methods were calculated for each questionnaire by ranking the total number of each rank for each method. Then the subjective ranks on general impression were matched with the objective ranks for individual clerk and the discrepancy was compared to determine the consistency of ranking between the two ranking systems. Statistical analysis and graphs have been completed by SPSS 17.0 and GraphPad Prism 5.0. Chi-square test and Analysis of Variance (ANOVA) were used for comparison of the three methods. Matched t-test was used for the comparison of objective ranking and subjective ranking.

## Results/Analysis

A total of 112 students from 16 batches have been enrolled into this study from September 2017 to May 2018. All these students were in their 4 ^th^ year of medical school and were trained as clerks in our department of neurology. 98 questionnaires were included.

### 3.2 Comparison with the ranks of the three methods

**Figure 2.**
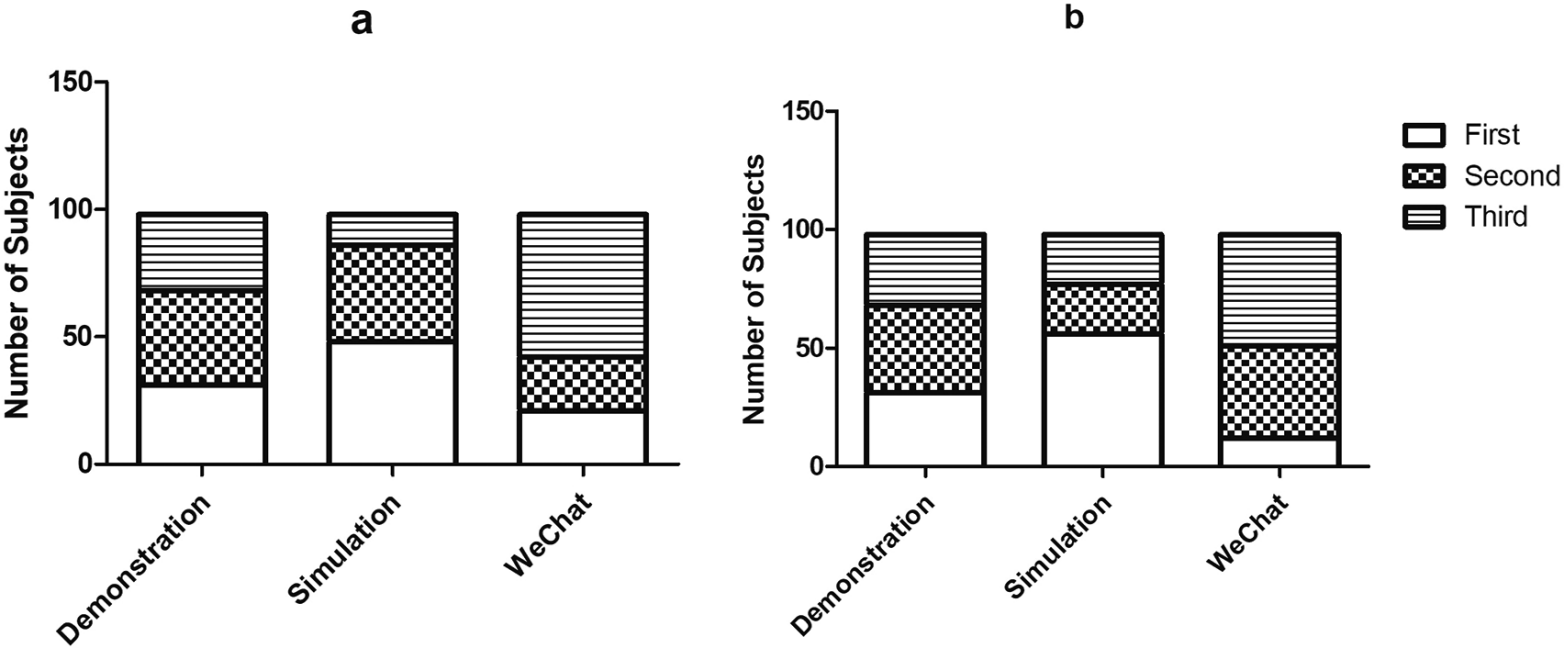
The general and overall ranking of the three teaching methods. Simulation via standard patients was considered the most popular methods according to either the general ranking (a) or the overall ranking (b).

Based on the general impression, about 49.0% participants thought simulation via standard patients was the best bedside teaching method for neurological clerkship, whereas 31.6% participants voted for modified bedside demonstration and only 21.4% participants favored WeChat-assisted teaching ( Figure 2a). The general ranks of the three methods were significantly different (P=0.000).

Then, the total number of ranks was counted respectively for the three methods under each item. As Table 1 shows, simulated patient teaching was ranked higher in comparison to either the modified bedside demonstration or WeChat-assisted teaching in terms of interpretation of theories, diagnostic skills, physical examination skills and communication skills (P＜0.05). It also provided better privacy protection (P=0.000) and provoked more interest in neurology among clerks (P=0.005). The modified bedside demonstration was found to benefit the performance in exams (P=0.009). All the three teaching methods were regarded as “well managed”, and ranked close to each other in terms of promoting knowledge, keeping participants confident and passionate (P> 0.05).

**Table 1.** Comparison of the three teaching methods in bedside teaching.

	Method 1 ^a^	Method 2 ^a^	Method 3 ^a^	P value
**Q1: How does the course improve your knowledge of disease processes?**				
**No. of rank: 1/2/3** ^b^	51/41/6	74/24/0	36/53/9	0.00 ^c^
**Q2: How does the course improve your knowledge of disease diagnosis?**				
**No. of rank: 1/2/3**	48/39/11	68/29/1	43/42/13	0.001 ^c^
**Q3: How does the course improve your knowledge of disease management?**				
**No. of rank: 1/2/3**	36/48/14	33/50/15	28/46/24	0.355
**Q4: How does the course improve your clinical skills (Physical examination)?**				
**No. of rank: 1/2/3**	44/42/12	63/32/3	23/44/31	0.00 ^c^
**Q5: How does the course improve your communication skills with patient?**				
**No. of rank: 1/2/3**	38/41/19	73/19/6	16/33/49	0.00 ^c^
**Q6: Have you treated patients with respecting privacy issues during the course?**				
**No. of rank: 1/2/3**	44/38/16	56/40/2	29/34/35	0.00 ^c^
**Q7: How does the course stimulate your interest in neurology?**				
**No. of rank: 1/2/3**	49/40/9	67/31/0	48/38/12	0.005 ^c^
**Q8: How does the course improve your scores in examination?**				
**No. of rank: 1/2/3**	53/37/8	45/45/8	31/48/19	0.009 ^c^
**Q9: How does the course help to keep you confident and passionate?**				
**No. of rank: 1/2/3**	38/48/12	54/39/5	43/43/12	0.141
**Q10: How do you feel about the bedside teaching course?**				
**No. of rank: 1/2/3**	82/13/3	85/12/1	71/20/7	0.096

*Abbreviations: No: number;*

^a^ *Method 1: modified bedside demonstration; Method 2: simulation via standard patients; Method 3: WeChat-assisted bedside teaching.*

^b^ *Total number of participants of rank 1,2 and 3, respectively.*

^c^ *P≤0.05.*

**Figure 3.**
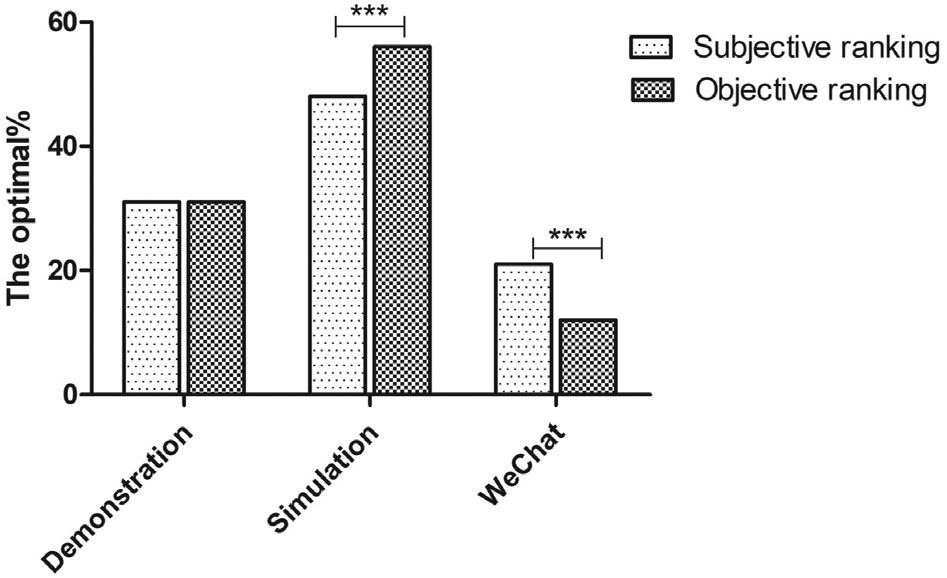
The proportion of a method vote as the best bedside teaching method in the general ranking and overall ranking. The percentage of being voted as the best bedside teaching method was significantly different for both simulation and WeChat-assisted teaching (P=0.000).

An overall rank was generated from the comprehension of respective ranks of each method. It was suggested that 31.6% questionnaire presented the modified bedside demonstration as the best teaching method, versus 57.1% supported simulation via standard patients and 12.2% approved WeChat teaching ( Figure 2b). The proportion of both simulated patient teaching and WeChat-assisted teaching as the best teaching method was significantly different between the general ranking and the overall ranking (P=0.000, Figure 3).

### 3.3 The difference between the subjective ranking and the objective ranking

**Figure 4.**
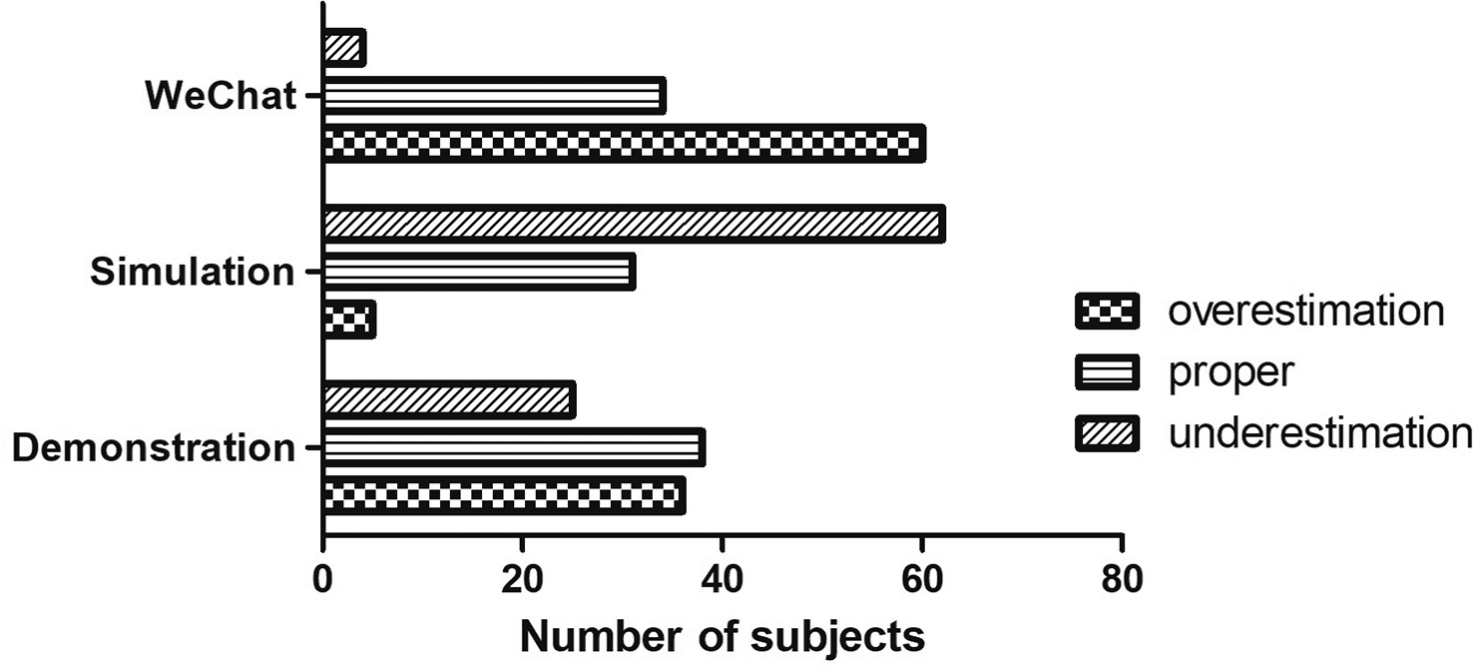
The variance of self-assessment among different teaching methods. The most overestimation was disclosed for WeChat-assisted teaching while the most underestimation was found for simulation among neurological clerks (P=0.000).

In accordance with the general ranking, we found simulation via standard patients was voted as the best bedside teaching method in the overall ranking. However, the consistency fluctuated between the subjective ranks based on general impression and the objective ranks derived from detailed assessment. The consistency of evaluation between the two ranking systems was 38.8%, 31.6% and 34.7% for the modified bedside demonstration, simulation and WeChat teaching, respectively. About 25.5% participants gave higher ranks to the modified bedside demonstration in the objective ranking system than in the subjective system, versus 63.3% to simulation and 4.1% to WeChat-assisted teaching. In contrast, 35.7% of the participants gave lower ranks to the modified bedside demonstration in the objective ranking system than in the subjective ranking system, versus 5.1% in simulation and 61.2% in WeChat-assisted teaching. The consistency of the two ranking systems was of significant difference ((P=0.000, Figure 4).

## Discussion

With increasing incidence of neurological diseases, it is more likely for all doctors, especially general practitioners and internal medicine physicians, to manage neurological patients. The clinical competency of initiating primary assessment and management of neurological diseases is essential for every graduating medical student ( Kandiyali *et al.*, 2017; Bradley, 2000). The difficulty in neurology learning elicits a negative impact to medical students on both the clinical competency of dealing with neurology patients and their career choice of becoming neurologists ( Pakpoor *et al.*, 2014).

Neurological clerkship has been documented as a valuable clinical rotation to alleviate or even eliminate the students’ fear of neurology and has a positive effect on neurology learning and career choice ( Albert *et al.*, 2015). For neurological clerks in the 4 ^th^ year of medical school in China, it is critical to integrate theories from textbooks into clinical skills. Real-life clinical scenarios should be included in the training during clerkship in order to ensure the smooth transition from preclinical students to clinical practitioners. Bedside teaching was a traditional but classic approach around the world to strengthen memory of theories and improve clinical competence. However, its popularity has dwindled since Boerhaave and Osler claimed for its major drawbacks due to limited teaching input of senior clinicians ( Qureshi, 2014; Verghese *et al.*, 2011). Nevertheless, bedside teaching continues to play an important role in the curriculum of neurological clerkship. It consolidates the repeated practice of physical examination on the nervous system, the competence of clinical reasoning about neuroanatomic diagnosis and qualitative diagnosis, as well as the management of common neurological diseases ( Roze *et al.*, 2018; Jayakumar, 2017). Besides, patients with neurological diseases usually present difficulties in communication and limited cooperation in history taking and physical examination, consequently increasing students’ fear of neurology. There was evidence that bedside teaching helps to overcome the fear and specifically enhance the skills of taking history, physical examination and counseling patients with impaired consciousness and yielded cooperation ( Peters and Ten Cate, 2014).

### 4.1 The preference of participants with the three bedside teaching methods

In this study, we investigated the attitudes and academic learning feedback of neurological clerks towards the three bedside teaching methods: modified conventional demonstration, simulated patient teaching and WeChat-assisted teaching. Most of the participants preferred simulated patient teaching according to either the general ranking or the overall ranking. Among the numerous evolving teaching methods in recent decades, simulation has been proved one of the most effective methods with the advantage of guarantying standardized learning experience ( Weller *et al.*, 2012). A recent study demonstrated that simulation-based education with deliberate practice significantly improved the confidence and clinical competence of students ( Offiah *et al.*, 2019). In addition to alleviating the fear of neurology, simulation-based education could also inspire the academic interest of students ( Galtrey *et al.*, 2018). Simulated patients have been considered as an effective way of clinical training by providing simulation of various neurological symptoms and signs of the real patients, as well as allowing repetitive clinical practice in a risk-free environment ( Galtrey *et al.*, 2018). In this study, the simulated patient was programmed to manifest typical symptoms and signs of three common neurological conditions: acute ischemic stroke, acute myelitis or myasthenia gravis. The simulated patient teaching provided more opportunities for clerks to practice clinical assessment and clinical reasoning at the bedside. Moreover, the simulated patients played by the experienced neurologist had the advantage of making the closest resemblance to the real patients, which improved students’ skills of communicating with patients from all walks of life. Compassion, empathy and patient confidentiality could also be promoted precisely during the simulation process without causing unnecessary disruption of clinical care of real patients or arousing excessive anxiety of the students when placed in the real conditions. By watching the performance of their peers and being assessed individually, the students received valuable feedback and became more actively engaged into learning. Thus, it is reasonable for simulated patient teaching to be the most popular one among the three bedside teaching methods.

Notably in this study, bedside teaching via WeChat did not achieve the expected outcome although the application of WeChat in medical education at several medical colleges in China has demonstrated satisfying outcomes in both problem-based learning and students-centered teaching ( Wang *et al.*, 2017; Zeng *et al.*, 2016b; Sun *et al.*, 2016; Zeng *et al.*, 2016a). All these studies are in accordance with another recent research showing that smartphones could be a significant learning tool for students as well as an addition instrument to academic teaching practice for staff ( Jabali *et al.*, 2019). However, these previous studies usually applied WeChat among junior medical students or residents. In the current study, WeChat was adopted for the senior graduating students in real clinic but proved to be less favorable for these participants with less desirable outcomes in either the general ranking or the overall ranking. Although some participants thought that WeChat teaching was as appealing as the other methods because it demonstrated the same effectiveness in terms of improving the management of disease, more participants stated that WeChat teaching was less beneficial than simulation or even modified bedside demonstration. The discrepancy with previous studies may be attributed to different target-students and inadequate utilization of its functions. Raising questions in private by WeChat was expected to encourage participants to disclose their drawbacks without adding embarrassment in front of their peers. At the same time, supervisors could assess the students by these questions and manage the teaching accordingly, as well as provide individualized guidance and make training more efficient and interesting. However, on analysis of the questions proposed, we found that the clerks were not so good at discovering key points of cases and proposing meaningful questions. The phenomenon may be attributed to the fact that the clerks were placed into real-life situation for the first time so that they were lack of the real-life experiences of clinical reasoning and critical questioning. On the other hand, WeChat takes advantages of facilitating active interaction, sharing information in multiple formats, and allowing instant assessment and feedback ( Sun *et al.*, 2016; Zeng *et al.*, 2016b). Unfortunately, the latter two functions were not fully explored in the present study. In spite of the increasing use of social medium to assist teaching, the feasibility of social medium like WeChat in China in clinical teaching need further exploration by targeting appropriate students and making good use of its advantages on assessment, feedback and information sharing.

In the traditional bedside teaching, students learn how to deal with sick patients from demonstration and illustration by their supervisors. Traditional bedside teaching takes the advantages of modeling teachers’ clinical assessment and reasoning directly for achieving the learning objectives ( Jayakumar, 2017). However, it rarely involves the discussion of clinical decision making. The information students received is scattered and hard to be integrated into a clear picture by students themselves. Therefore, poor learning outcomes in terms of the construction of clinical thinking system, and clerks may still present with performance anxiety or even phobia after training when dealing with a real-life patient waiting for the instant diagnostic and management. We previously made improvement for the traditional bedside teaching by adding group discussion and illustration in classroom after bedside demonstration, promoting the teaching effectiveness in neurological clerkship ( Jingjing *et al*., 2017). However, in the present study, the modified bedside demonstration was ranked less popular than simulated patient teaching. First, the clerks were less engaged in the modified bedside demonstration than in the simulation. Second, less opportunity of practice in the modified bedside demonstration negatively affected the transition from knowledge to skills. Nevertheless, most of the participants tended to believe that the modified bedside demonstration could guarantee excellent performance in exams, which might indicate that assessment guideline in the current system has put more emphasis on knowledge rather than skills.

### 4.2 Difference between the subjective rank and objective rank

In our study, subjects were asked to rank the three teaching methods according to their general feeling about the courses and detailed items respectively. The objective ranks derived from the ranks of separate items reflects the academic assessment of the three methods after careful thinking of students with the tips of detailed questionnaire, while the subjective ranks (the general ranks) of the three methods shows the emotional impression of students towards the methods.

The difference between the subjective rank and the objective rank is considered to reflect the gap between the participants’ feeling about the course and the final critically-appraised reflection on their learning in the course. The ideal result is that the recall about the course in general could stay consistent with the recall reflecting detailed assessment. When the rank of a certain method in the subjective ranking exceeded it in the objective ranking, it is considered the clerks did not feel so satisfied in the academic assessment as they felt in the emotional assessment. In other words, the effectiveness of the method was overestimated and a higher rank was given when the subject evaluated his or her performance during teaching with the method according to the general impression. However, the rank declined when the subject thought about the learning outcomes carefully over the detailed items. The decrease of ranks from subjective ranking to objective ranking suggested that course coordinators should make the course easier or provide more positive feedback to inspire more confidence and enthusiasm of students. Conversely, when the clerks thought they performed poorly with a certain teaching method for the emotional impression but committed actually academical promotion, they tended to give an unfavorable reputation in general but a better comment in detailed evaluation. In this condition, the rank in subjective assessment was beneath it in the objective assessment, suggesting subjects underestimated themselves according to the general impression. Accordingly, a more profound course could be explored and adequate training should be included to achieve the teaching outcomes.

Interestingly, although participants in this study reported equal confidence and enthusiasm for the three methods in detailed assessment, the subjective ranks of the three methods were not in complete accordance with the objective ranks, suggesting that sometimes the influence of teaching methods on self-assessment was unaware even by students themselves. For both simulation course and demonstration course, only about one third subjects presented with consistent results between general evaluation and detailed assessment. The highest percentage of underestimation of oneself was found in simulation, which was up to 63.3%. The fact that more students underestimated themselves in simulation may be attributed to the more mistakes associated with more simulated case exposure and more opportunities of practice. Students may feel frustrated and awkward in front of teachers and peers when mistakes occur, leading to reduced clinical confidence during their clerkship. As known, clinical confidence is important in achieving the best educational outcome for medical students. There has been ample evidence showing that how the students feel about their performance in clinical practice would potentially have a significant impact on how they would practice medicine in their future clinical career ( McNair *et al.*, 2016). Therefore, the apparent biased self-assessment for the general impression could negatively affect the learning attitudes and outcomes. Instant supervision and feedback must be included during teaching, and intervention should be taken when necessary to ensure teaching is in a positive learning cycle of feedback, reflection and action. Simulation used to be established to provide students with a motivating and engaging learning process ( Weller *et al.*, 2012). However, the results of the current study indicate that simulation could lead to underestimation of oneself. Therefore, the teaching must be progressive, and positive feedback should be appropriately given to maintain the ongoing interest and engagement of students.

Likewise, the most proportion of participants showing excessive confidence in WeChat-assisted teaching may also be explained by the fact that WeChat-assisted teaching could make the participants’ training less challenging and stressful. Clinical materials assigned before the teaching might help participants prepared for the coming challenges, which would be less predictable in real-life clinic. Moreover, participants who could browse answers on their phones are less stressful and are more probable to overestimate themselves. WeChat was introduced into bedside teaching during neurological clerkship with an expectation that it could diminish the neurophobia of students in front of their peers and real-life patients. Then the students could focus on learning process and improve the learning efficiency. However, it suggested that bedside teaching assisted by social medium like WeChat need to be well-designed to avoid biased self-assessment while making good use of its advantages.

This well-designed study has provided every participant with all the effective teaching models in order to ensure that participant experiencing all three methods could give a relatively objective assessment. The random order of the three teaching methods eliminated potential bias in view of the participants’ developing capability during training. Limitation of this study lies in the lack of comparison based on the formal objective assessment such as the objective structured clinical examination (OSCE) format, which we are planning to be included in further studies.

## Conclusion

For both the general ranking and the overall ranking, simulated-patient teaching is the most favorable and effective bedside teaching method for neurological clerks to promote clinical competence, in comparison to the modified bedside demonstration and WeChat-assisted teaching. Clerks tended to underestimate themselves in simulation so positive feedback should be given appropriately. WeChat-assisted bedside teaching for neurological clerks needs further exploration to delivery effective teaching and minimize self-overestimation.

## Take Home Messages

• Simulation is the most popular bedside teaching method among neurology clerks compared with demonstration and WeChat-assisted teaching.
• Simulated patients facilitate the bedside teaching of neurology clerkship training in terms of attitude and competence.
• WeChat-assisted teaching should target appropriate students and make good use of its advantages on assessment, feedback and information sharing.
• Simulation could lead to underestimation of oneself whereas WeChat-assisted teaching could lead to overestimation of oneself, suggesting that the instant adjustment of teaching is necessary to ensure the ongoing interest and engagement of students.

## Notes On Contributors

**Hongyan Zhou**: Dr. PhD., Associated professor, Department of Neurology, the First Affiliated Hospital of Sun Yat-sen University, Guangzhou, China.

**Li Feng**: Dr. PhD., Attending, Department of Neurology, the First Affiliated Hospital of Sun Yat-sen University, Guangzhou, China. ORCID: https://orcid.org/0000-0001-6724-7153

**Yaru Lu**: Dr., Resident, Department of Neurology, the First Affiliated Hospital of Sun Yat-sen University, Guangzhou, China.

**Jingjing Li**: Dr. PhD., Associated professor, Department of Neurology, the First Affiliated Hospital of Sun Yat-sen University, Guangzhou, China.

**Xunsha Sun**: Dr. PhD., Attending, Department of Neurology, the First Affiliated Hospital of Sun Yat-sen University, Guangzhou, China.

**Cunzhou Shen**: Dr. PhD., Attending, Department of Neurology, the First Affiliated Hospital of Sun Yat-sen University, Guangzhou, China.

**Weixi Zhang**: Dr. PhD., Professor, Associated chief of Department of Neurology, the First Affiliated Hospital of Sun Yat-sen University, Guangzhou, China.

**Ling Chen**: Dr. PhD., Professor, Department of Neurology, the First Affiliated Hospital of Sun Yat-sen University, Guangzhou, China. ORCID: https://orcid.org/0000-0002-6909-9606

**Dan Xu**: Dr. PhD., Professor, Department of General Practice, Dean of Curtin Medical School, Faculty of Health Sciences, Curtin University, Australia.

**Ming Kuang**: Dr. PhD., Professor, Zhong-Shan School of Medicine, Department of Hepatic Surgery and cancer center, Institute of Precision Medicine, Associated administrator of the First Affiliated Hospital of Sun Yat-Sen University, Guangzhou, China.

**Huiyu Feng**: Dr. PhD., Professor, Department of Neurology, the First Affiliated Hospital of Sun Yat-sen University, Guangzhou, China. ORCID: https://orcid.org/0000-0003-1153-5919

## Declarations

The author has declared that there are no conflicts of interest.

## Ethics Statement

The research was conducted in accordance with the Declaration of Helsinki, and was approved by the ethical committee of the First Affiliated Hospital of Sun Yat-sen University.

## External Funding

This research was supported by The Southern China International Cooperation Base for Early Intervention and Functional Rehabilitation of Neurological Diseases (2015B050501003); The undergraduate teaching reform project of SYSU: (80000-18832619, 31911130-200433, 31911130-200435) and Project of Guangzhou Science Technology and Innovation Commission (201707010122).
