# Supplementary material for: What is the best option to manage the bedside teaching for Neurology Clerkship -- Demonstration, Simulation or WeChat teaching?
Source: MedEdPublish (2016). 2020 Dec 22;9:286. [Version 1] doi: 10.15694/mep.2020.000286.1 (PMC10698730; doi:10.15694/mep.2020.000286.1)
Supplement: Peer review history

# Reviewer report: Reviewer response for version 1

**DOI:** 10.21956/mep.20080.r30903

**Published:** 2021-01-10

**Authors:** Yingzi Huang

**Affiliations:** 1 The First Affiliated Hospital, Sun Yat-sen University

This review has been migrated. The reviewer awarded 4 stars out of 5 I enjoyed reading this interesting article comparing the three types of clinical teaching, although simulation without providing real patient is not supposed to be one of the types. Wechat learning is a more popular type now in China especially in the context of the pandemic, thus it provides valuable insights for those who are interested in relevant teaching. And it could be more insightful if combined with a conceptual framework of activity theory or even change laboratory theory for improvement in the real world, especially when it comes to students’ fear of neurology. Thus the title could have been switching away from only aiming at the best option for a complex issue, as there is perhaps no best option but better solutions and improvements. Students’ fear of neurology is a much more complex issue than one that can be tackled only through providing options of bedside teaching during clinical clerkship. It might involve defining core competences, integration of basic medicine and clinical medicine in earlier years in undergraduate level. Were they on the same level before they entered the project and should there be a baseline assessment of their competence, considering students in the same year in clerkship still may differ in their respective competences. Feedback is an essential element both in traditional clinical teaching and simulation teaching, thus it’s less likely to be the reason for students’ underestimation of their own competence, unless if the feedback was provided inadequately improperly in the first place. It’s bit confusing to see the questionnaire’s item writing with all open questions starting with ‘how’ and the results are ranking of numbers. It seems then useful to provide the response options to figure out the meaning of the results.

Reviewer Expertise:

NA

No decision status is available

# Reviewer report: Reviewer response for version 1

**DOI:** 10.21956/mep.20080.r30904

**Published:** 2021-01-05

**Authors:** Balakrishnan Nair

**Affiliations:** 1 Centre for Medical Professional Development and University of Newcastle

This review has been migrated. The reviewer awarded 4 stars out of 5 "Neurophobia " is a well-known phenomenon among medical students and junior doctors. This is because clinical skills in neurology is not well taught in medical schools. The only way to improve the situation is to provide good quality teaching. So this article is important from that point of view. They have used role players. The traditionally the accepted view is "bedside teaching" is when there is a real patient present. The title may need to change to reflect this .Otherwise, this is a good study

Reviewer Expertise:

NA

No decision status is available

# Reviewer report: Reviewer response for version 1

**DOI:** 10.21956/mep.20080.r30905

**Published:** 2021-01-02

**Authors:** BALAJI ARUMUGAM

**Affiliations:** 1 TAGORE MEDICAL COLLEGE AND HOSPITAL

This review has been migrated. The reviewer awarded 4 stars out of 5 Dear authors, The well conducted study compared the three modalities of TL methods.Its an educational interventional study with the objectives of comparing simulation, bedside demonstration and Wechat assisted teaching. The results suggested that simulated patient teaching was ranked higher in comparison to either the modified bedside demonstration or WeChat-assisted teaching in terms of interpretation of theories, diagnostic skills, physical examination skills and communication skills.The modified bedside demonstration was found to benefit the performance in exams. All the three teaching methods were regarded as “well managed”, and ranked close to each other in terms of promoting knowledge, keeping participants confident and passionate. Note/ Suggestion: Wechat assisted teaching needs more clarification because it included social media chatting, follow up and also bedside clinic - two methods were followed but showed very less importance in the way of learning among the study participants.

Reviewer Expertise:

NA

No decision status is available
